# New Perspectives on Burnout: A Controlled Study on Movement Analysis of Burnout Patients

**DOI:** 10.3389/fpsyg.2018.01150

**Published:** 2018-07-09

**Authors:** Manuela M. Pfeffer, Andrea Paletta, Gerald Suchar

**Affiliations:** ^1^Institute of Sport Science, University of Graz, Graz, Austria; ^2^Privatklinik St. Radegund, St. Radegund, Austria

**Keywords:** burnout, body movement, body language, Laban Movement Analysis, effort

## Abstract

**Introduction:** Despite extensive research on burnout, there has been to date no systematic movement analysis of burnout patients, although it is well known that psychiatric diseases express themselves through movements, such as psychomotor retardation or agitation. Since the movement expression of burnout patients has not been systematically investigated so far, the aim of this study is to close this knowledge gap in order to obtain a new perspective on burnout.

**Methods:** Hospitalized burnout patients (*n* = 22; age 47.2 ± 9.1 years) and health controls (*n* = 20; age 41.5 ± 15.0 years) participated in a standardized movement sequence with verbal instructions. The objective Burnout Inventory Scale and diagnostics by psychiatrists were used for diagnosis. Two certified movement-analysts independently rated each participant via video by using the Effort System of Laban Movement Analysis as an instrument of dance therapy and behavior observation. Cohen’s Kappa was used to test the inter-rater reliability of the movement analysts and non-parametric Mann–Whitney *U* tests were undertaken to assess the differences between the two groups.

**Results:** The rater-agreement Kappa ranges from 0.66 to 0.92 (*p* < 0.001) with the Confidence Interval (95%) from 0.46 to 1.1. Results of the Mann–Whitney *U* tests indicate that burnout patients show significantly less frequent movements for the following Effort elements: Bound *U*(*n*_1_ = 22, *n*_2_ = 20) = 112.5, *p* = 0.001; Indirect *U*(*n*_1_ = 22, *n*_2_ = 20) = 114.5, *p* = 0.001; Light *U*(*n*_1_ = 22, *n*_2_ = 20) = 115, *p* = 0.001 and Sustained *U*(*n*_1_ = 22, *n*_2_ = 20) = 130, *p* = 0.01.

**Discussion:** Burnout patients have significant deficits in all four Effort elements of the Laban Movement Analysis (Flow, Space, Time, Weight) and therefore have deficits regarding their body movement. The findings presented here provide an additional perspective on burnout.

## Introduction

‘Burnout’ was first mentioned by Freudenberger in 1974 and Maslach in 1976 to describe the problems of exhausted employees in the health services (Schaufeli et al., 2009). In the current version of the International Classification of Diseases, ICD-10 (World Health Organization, 2016), burn-out (Z73.0) can be found under State of vital exhaustion (Z73) ‘problems related to life management difficulty’ excluding ‘problems related to socioeconomic and psychosocial circumstances’ (Z55–Z65). It is to be found under ‘factors influencing health status and contact with health services’ (XXI). Thus, according to the ICD-10, burnout is not recognized as a discrete disorder, as it is neither in the current Diagnostic Manual of Mental Disorders, DSM-5 (American Psychiatric Association et al., 2014). Burnout is widely seen as a chronic stress-related syndrome with the three dimensions exhaustion, cynicism, and inefficacy (Maslach et al., 1996, 2001; Schaufeli and Enzmann, 1998; Maslach, 2003, 2004; Maslach and Leiter, 2008) or as physical, emotional and mental exhaustion because of chronic emotionally demanding work (Schaufeli and Greenglass, 2001). Within the main concepts of burnout, exhaustion is the major symptom (Cox et al., 2005).

According to epidemiological studies burnout has not only appeared within health services (Freudenberger, 1974; Maslach, 1976; Maslach et al., 1996; Maslach and Schaufeli, 2017), but also within other occupations, such as clerical, military, computer technology and business as well as in non-occupational areas, such as political activism and within the family (Maslach et al., 2001; Maslach and Schaufeli, 2017). Furthermore, it has also been shown to be prevalent in both industrialized as well as in developing countries (Carod-Aartal and Vázquez-Cabrera, 2013). In Austria, a recent study indicated that the prevalence among a cross-section of the population is around 8% (Scheibenbogen et al., 2017). They further showed that especially the groups under 30 years and between 50 and 59 years are at high risk of being affected. However, no differences regarding gender were found.

Given the aforementioned evidence of burnout being epidemic, it can be seen that it is associated with widespread burdens placed on individuals, organizations and societies. Just as Bianchi et al. (2015a) have demonstrated, burnout has been related to absenteeism (Ahola et al., 2008), presenteeism (Demerouti et al., 2009), poorer work performance (Taris, 2006), job turnover (Leiter and Maslach, 2009), chronic work disability (Ahola et al., 2009b) and disability pensions (Ahola et al., 2009a). Moreover, burnout has been shown to precipitate severe injuries (Ahola et al., 2013), insomnia (Armon et al., 2008), hospitalization for mental and cardiovascular disorders (Toppinen-Tanner et al., 2009), coronary heart disease (Toker et al., 2012) and is associated with accelerated biological aging (Ahola et al., 2012) and all-cause mortality (Ahola et al., 2010). In addition to the health consequences, the economic consequences are also rapidly emerging. Direct and indirect financial costs are emerging not only for employers and organizations but also for nations (Carod-Aartal and Vázquez-Cabrera, 2013; Schneider and Dreer, 2013). Therefore, investigation into burnout is of great public and economic interest.

Despite the fact that burnout is highly prevalent and associated with consequences for individuals, organizations and societies, there is still no international consensual diagnostic criteria for burnout (Weber and Jaekel-Reinhard, 2000; Bianchi et al., 2015a; Van Dam, 2016). The most widely used and evaluated instrument to measure burnout within the scientific community is the Maslach Burnout Inventory (MBI) (Maslach and Jackson, 1981), which has been translated into different languages, used in different professions and cultural contexts and which has also been criticized methodologically (Worley et al., 2008; Aguayo et al., 2011; Burisch, 2014). Aguayo et al. (2011). Not least the MBI is criticized because of its sole focus on job-relation and because of it lacking thresholds to diagnose burnout (Lehofer et al., 2011). The absence of international consensual diagnostic criteria has led to the application of various criteria in research (Bianchi et al., 2015a). Until now, the burnout construct has been questioned (Bianchi et al., 2015a) regarding the scope (related to work vs. without context), cardinal symptoms (e.g., inclusion of cognitive impairment), process (onset, offset, duration, relapse), structure (uni- vs. multi-dimensional) and distinctiveness (Schaufeli and Enzmann, 1998; Cox et al., 2005; Kristensen et al., 2005; Hakanen and Schaufeli, 2012; Bianchi et al., 2015a). The distinctiveness of depressive, adjustment, anxiety or fatigue disorders is also questioned (Ahola et al., 2005, 2014; Toker et al., 2005; Korczak et al., 2010; Bianchi et al., 2014, 2015a,b; Van Dam, 2016).

The continuing lack of international consensual diagnostic criteria and also lack of differential diagnostics imply of course certain problems and limitations for burnout research and therefore also for this investigation. However, because it is not the aim of this study to clarify these important and basic questions, this study can only begin at a certain point, but with the knowledge of the underlying discussions and therefore also with these underlying limitations. So, the diagnosis of burnout of this investigation is limited to the diagnosis of the psychiatrists of a clinic as well as a German burnout questionnaire with good measurement properties, that also tests resources, and which is not only job-related (BOSS; Geuenich and Hagemann, 2014). The underlying burnout concept of this investigation corresponds to the main concepts of burnout, that exhaustion is the major symptom of burnout (Cox et al., 2005).

In terms of extensive research on burnout, to the best of our knowledge, no movement analyses of burnout patients have been undertaken, whereas there are several for other psychiatric diseases. This can provide a new perspective on burnout. Based on the bio-psycho-social medical model, the investigation of all three perspectives should strive to effectively tackle medical problems. Such an approach promises additional and deeper knowledge of the phenomenon. Thus, besides the psychological investigation, a psychiatric disease also needs a social and biological perspective. In this model, the biological perspective includes biological and physiological markers as well as phenomenological descriptions of bodily sensations, such as body movement expression. According to this demand, it is well known that psychiatric diseases have, in fact, their expression in movements, e.g., psychomotor retardation or agitation, which are listed in the disease classification systems (ICD-10, DSM-V). However, to the knowledge of the authors, the movement expression of burnout patients has not yet been systematically investigated.

To investigate the movement expression of patients, there are various types of analysis systems. However, for this study the Laban Movement Analysis (LMA), which is described in Laban and Lawrence (1947); Bartenieff and Lewis (1980), Laban (2003, 2011), Bender (2010), and Kennedy (2014), was used for several reasons. The LMA is well established with its systematic language for human movement. It is descriptive, neutral and objective as well as precise, specific and mathematical (Foroud and Whishaw, 2006; Masuda and Kato, 2010; Lausberg, 2012). It is the diagnostic instrument in dance and movement therapy (Bartenieff and Hackney, 1984; Lausberg, 1997, 2012) and is a standardized scientific method for movement observation (Lausberg, 1997, 2012; Welsche et al., 2007). In addition, the LMA has been applied in experimental research (Foroud and Pellis, 2003; Whishaw et al., 2003; Foroud et al., 2004), and is used in neurosciences for analyzing movement behavior (Foroud and Whishaw, 2006) as well as in research regarding animation techniques, robotics and surveillance techniques (Rett et al., 2008; Masuda and Kato, 2010; Kim et al., 2013). Furthermore, the LMA covers the quality, i.e., the expressive and non-kinematic specificities of a movement, and not only the quantitative and kinematic characteristics of a movement behavior (Levy and Duke, 2003; Foroud and Whishaw, 2006; Lausberg, 2012; Samadani et al., 2013). The qualitative and non-kinematic aspects of the movements are particularly interesting when investigating a psychiatric syndrome. As it deals with the specific phenomenology of movement, non-kinematic abnormalities in movements such as fatigue, increases in effort or intrusive movements can be documented. However, they cannot be documented with kinematic or biomechanical analyses (Foroud and Whishaw, 2006). For this study the movement of the participants was analyzed with the LMA Effort System (see section “Materials and Methods”).

The Effort System is one definite part of the LMA. A matter of interest of the Effort is the intensity of the movement (Foroud et al., 2004) or “the change in the intensity of exertion throughout the movement” (Foroud and Whishaw, 2006). So, exhaustion could be seen as the absence of Efforts. Therefore, it seems natural to analyze at least the LMA Effort System in connection with burnout patients, since in the main burnout concepts exhaustion is the major symptom of burnout (Cox et al., 2005). The Effort System with its elements was derived from the external motion factors space, time, gravity/weight and flow (continuous ongoing vs. completely stopping). The Effort elements Space, Time, Weight and Flow range between two types of Effort qualities: from indulging to fighting against the external motion factors. The indulging elements do not resist (although they are active qualities), while the fighting elements move against the motion factors. The indulging and fighting qualities within one Effort element cannot be shown simultaneously because one either indulges in an external motion factor or one is fighting against it. However, the indulging and fighting qualities of different Effort elements can be shown simultaneously. It can be also show more Effort elements simultaneously or an absence of all Effort elements throughout a movement. (Laban and Lawrence, 1947; Bartenieff and Lewis, 1980; Laban, 2003, 2011; Bender, 2010; Kennedy, 2014).

There have been several scientific investigations in which the LMA was used to analyze the movements of patients with psychiatric diseases such as anorexia nervosa (Burn, 1987; Shenton, 1990; Lausberg et al., 1996; Lausberg, 1998), bulimia (Lausberg et al., 1996; Lausberg, 1998), depression (Welsche, 2010), schizophrenia (Cruz, 1995), borderline personality disorder (Degener et al., 2011), other personality disorders (Cruz, 1995), and psychosomatic disorders (Lausberg et al., 1996). Using the Effort System, Burn (Burn, 1987) investigated anorectic patients and found within the Effort Flow fewer ‘Free’ movements and within the Effort Time more ‘Sustained’ movements in comparison to a healthy control group. Shenton (1990) found more ‘Bound’ movements within the Effort Flow. In addition, Shenton found fewer ‘Strong’ movements within the Effort Weight and a skewed usage of Space and Time. In comparison to a heterogenic group of psychosomatic disorders Lausberg et al. (1996) observed that anorectic patients showed more ‘Sudden’ movements. Patients with anorexia nervosa, bulimia and inflammatory bowel disease showed significantly fewer Strong movements within the Effort Weight as well as fewer Free movements within the Effort Flow in comparison to a healthy control group. However, there were no significant differences between the diseases whatsoever (Lausberg, 1998). Patients with depression showed on average a smaller repertoire relative to the Effort System. Moreover, they showed a preference for the fighting Effort Elements, which are Bound, Direct, Strong and Sudden (Welsche, 2010).

The movement of burnout patients has, to the knowledge of the authors, not been investigated yet, neither with the LMA nor with any other movement analysis system. Therefore, the aim of the study is to close this knowledge gap in order to obtain a new perspective on burnout. The focus is not on bodily symptoms, such as vegetative dysregulations, but to investigate these equivalents in terms of body expression and movement in relation to burnout. So, the aim of the study is to analyze the movement of burnout patients, and to closely analyze the movement expression of burnout patients in comparison to healthy people with the LMA Effort System to detect possible deficits. It is our opinion that the link between movement expression and burnout might be important for several reasons. Firstly, an understanding of movement expression of burnout patients could lead both to an additional perspective for investigating the syndrome, and to an extension of diagnostic advice in the future. Secondly, potential deficits within the movement expression of burnout patients can give new indications for movement interventions in the treatment of burnout. Thirdly, the potential deficits within the movement expression of burnout patients may give new starting points for preventive movement programs in health promotion. The findings in the studies of LMA with psychiatric patients, as well as a pilot study, suggest that burnout patients have deficits in their movements regarding the LMA Effort System. Indications were already given by the literature about burnout definition which considers exhaustion to be the relevant symptom (Maslach et al., 1996; Schaufeli and Enzmann, 1998; Brenninkmeijer and Van Yperen, 2003; Bekker et al., 2005; Cox et al., 2005; Kristensen et al., 2005; Van Dam, 2016). The research question of this study is: Do burnout patients have deficits within their movement in comparison to a healthy control group, analyzed with the LMA Effort System? It was hypothesized that burnout patients show deficits in their movements regarding the LMA Effort System. Moreover, it was hypothesized that the deficits affect all four Efforts of the Effort System.

## Materials and Methods

The ethical approval for the study was obtained from the Ethical Committee of the University of Graz (GZ. 39/5/63 ex 2014/15, 08.01.2015).

### Participants and Procedure

#### Participants

Twenty-three burnout patients (14 male and 9 female), hospitalized in a rehabilitation clinic for psychiatric illnesses, were tested. The control group consisted of 21 participants (10 male and 11 female). Two participants were dropped after the test because of the diagnosis of burnout by means of a burnout questionnaire. So, 22 burnout patients (14 male and 8 female, age 47.2 ± 9.1 years) and 20 healthy subjects (10 male and 11 female, age 41.5 ± 15.0 years) were analyzed. A comparable age-distribution across the two groups was sought to avoid influences due to age related changes in movements. Thus elderly people (over 65 years) and non-mature/young adults (under 25 years) were excluded.

Additionally, data regarding educational level, dance affinity, dance and dance therapy experience, as was well intake of medication with a five-tier Likert scale was gathered because these variables were quite likely to influence the movement. Education level was then defined as follows: low = below university entrance diploma, middle = university entrance diploma, high = higher than university entrance diploma. 13 burnout patients have a low, 5 a middle and 4 a high education level. In the control group 4 participants have a low, 6 a middle and 10 a high education level. 11 burnout patients have a dance affinity and 3 have dance (therapy) experience, whereas 12 participants in the control group have a dance affinity and 8 have dance (therapy) experience. All burnout patients took psychotropic drugs, and none were taken by the control group. Burnout patients took sedating as well as stimulating psychotropic drugs, often together. As we know, sedatives and stimulants can affect movement (see section “Discussion”).

Between their 1st and 2nd week of hospitalization the burnout patients were asked by the attendant psychiatrists in the clinic to participate in a scientific study. The control group was recruited through social media and mailing lists. If they agreed to participate they were informed about the study and they signed a consent form. The participants received only basic information about the procedure of the study in which they were involved. Regarding the movement session, they only received information that it would be a session with very easy, not exhausting movement tasks to avoid dance or movement affinity with the participants.

Inclusion criteria for the burnout patients was the diagnosis of burnout. Exclusion criteria for the subjects of the control group was the diagnosis of burnout. Exclusion criteria for all participants were other psychiatric conditions besides burnout and somatic illnesses or intellectual impairment, as this could influence movement behavior. All participants were blind to the aim, the hypothesis and the different participant groups.

### Measures

#### Burnout

All participants were asked to fill out the German versions of the Burnout Screening Scales (BOSS I–III) (Geuenich and Hagemann, 2014). The BOS-Scales I are about: job (10 items), self (10 items), family (5 items), friends (5 items). With the *job* scale, data about discomforts regarding one’s own job, described on a cognitive, emotional and behavioral level, is collected. The *self* scale is about the person, individuality and the individual’s overall situation. Aspects of the body, emotion and cognition are investigated along with it. On the *family* scale, conflicts and tendencies of neglect in one’s own family are included. With the *friends* scale, tendencies toward social withdrawal and inner isolation as well as tensions with friends are considered. The BOS-Scales II concern: physical problems (10 items), cognitive problems (10 items) and emotional problems (10 items). The *physical problems* scale is addresses several physical paresthesias and pain, especially regarding the cardiovascular, respiratory, digestive and immune systems as well as general parameters such as sleep quality. With the *cognitive problems* scale discomforts in terms of concentration, control of attention, perfectionism, decisiveness, rumination, etc. are studied, in other words mental conditions and processes which are often connected with chronic stress. With the *emotional problems* scale, data about specific emotions like anxiety, shame and mistrust are collected. Furthermore, emotional attitudes regarding one’s own person (e.g., confidence) and behavior patterns (e.g., withdrawal) are a matter of interest. The BOS-Scales III are concerned with satisfaction and resources in these areas: job (5 items), self (5 items), family (5 items), and friends (5 items). With the *job* scale meaningfulness, appreciation, affiliation, communication with colleagues and identification with one’s own job are investigated. The *self* scale considers self-evaluation, self-esteem and homeostasis. With the *family* scale data regarding nearness, emotional security, appreciation, reciprocal acceptance and concern for one’s own family and/or partnership is collected. Lastly, with the *friends* scale, concern, trustworthiness, acceptance, affiliation und respect amongst one’s own friends are examined. So, the BOSS also concerns resources and is not only job-related (Geuenich and Hagemann, 2014).

Validity of the BOSS: Besides correlations with other questionnaires, the questionnaire shows good criterion-related validity as the statistical values coincide with the clinical diagnosis of psychiatrists (Geuenich and Hagemann, 2014).

Thus the burnout diagnosis by the psychiatrists in the clinic was further confirmed by the questionnaire *Burnout Screening Scales* (BOSS I–III) (Geuenich and Hagemann, 2014), which was the inclusion criteria for the hospitalized burnout patients. Patients suffered from *clinical burnout*, which means that they were hospitalized, diagnosed by a clinician with burnout, unable to work and receiving psychological treatment (Schaufeli et al., 2001; Brenninkmeijer and Van Yperen, 2003; Oosterholt et al., 2014; Van Dam, 2016). Differential diagnostics (depression, anxiety or fatigue disorders etc.) were not tested by means of additional psychological tests but were implicit with respect to the psychiatric diagnoses from the psychiatrists at the clinic and the burnout questionnaire. So, a possible comorbidity between burnout and depression – as it is discussed in literature – was not clarified through additional psychological tests. Inclusion criteria for the control group were normal (i.e., healthy) values for this questionnaire. One participant from the patient group and one participant from the control group were dropped because one of the burnout patients had normal (i.e., healthy) values in the burnout questionnaire and one of the healthy control group displayed unhealthy (burnout) values.

#### Setting

The participants were divided into several burnout groups and healthy groups (5 groups, *n* = 10 ± 2) to simplify the test procedure. The distribution of the burnout patients and the healthy participants in their groups was random. Burnout patients and healthy participants were not mixed together to avoid influencing their movement behavior. The participants were asked to move according to verbal movement instructions. These movement sequences took place in the gym at the rehabilitation clinic (Privatklinik St. Radegund) between 2 and 3 pm. Five cameras filmed the movements from five different angles. A dance therapist, trained in LMA, gave the verbal movement instructions. She was blind to the hypothesis and excluded from the later test evaluation in order not to influence the participants or the analysis. In addition, the researchers were present as silent observers.

#### Movement Instructions

The standardized movement instructions consisted of three parts: A warm up (10 min) to become familiar with the room, the situation and the cameras, and to ensure that normal movement behavior was achieved. The second part (20 min) consisted of simple movement instructions such as “Please move at a comfortable tempo.” The third part (20 min) contained the invitation to improvise the four elements: earth, water, fire, air; it was oriented on Lausberg (1998), such as “Please try to represent earth with your movements.”

#### Movement Analysis

In order to test the hypothesis, the Flow, Space, Time, and Weight Efforts of the hospitalized burnout patients and of the healthy control group were analyzed using the LMA method by two independent movement analysts (experts in LMA) and these were compared. They analyzed each participant via video independently from each other as well as independently from the dance therapist who gave the verbal instructions, and from the researchers. They were blind to the distribution of the participants in the groups, the aim and the hypothesis.

#### Variables: The Flow Effort

The Flow Effort is about the continuity and the progress of the movement. The indulging quality of the Flow Effort is Free Flow: the movement is free of tension so that the antagonist muscles resist as little as possible during a movement, and therefore the movement cannot be stopped at any time. To shake out or swing the arms has the quality of Free Flow. The fighting quality of the Flow Effort is Bound Flow: the movement is controlled with increased tension so that not only are the agonists or active muscles contracted, but also the antagonists during a movement therefore can be stopped at any time. Ballet or Tai Chi have the quality of Bound Flow (Bartenieff and Lewis, 1980; Foroud et al., 2004; Bender, 2010; Kennedy, 2014).

#### Variables: The Space Effort

The Space Effort is about the attention given regarding the space and the objects within it. It describes the how, but not the wherefore. The Space Effort describes – with the indulging quality being Indirect and the fighting quality being Direct – whether the movements are oriented directly or indirectly with regard to the surroundings or objects. All team games need a lot of Indirect: the players need to overview the court and recognize their own team members without facing them with an encompassing focus. Only when it comes to shooting does the player’s concentration have the quality of Direct and is focused on one point. If there is no interest in the space whatsoever – neither a direct nor an indirect one, the person is simply moving – the Effort element Space is not present (Bartenieff and Lewis, 1980; Bender, 2010; Kennedy, 2014).

#### Variables: The Time Effort

The Time Effort is about the time, but not the duration of a movement or the objective measure of the time. It is about how one approaches whatever the duration of time is. One can either have the feeling of fighting against the motion factor time or indulging it. The indulging quality of the Time Effort is Sustained. Sustained means to have all the time in the world, so one can take one’s time to accomplish movements. The fighting quality of the Time Effort is Sudden/Fast. Sudden/Fast movements are used to move objects or one’s self by means of acceleration. It is not only about the higher tempo of the movement but also about fighting against the time, such as an old man with mobility problems crossing the street who tries to fight against the time that the light will remain green. For example, if a fit jogger moves faster and outdistances the old man, the jogger does not fight against the time the light is green with accelerated movements but has all the time in the world to cross the street and moves therefore within the Sustained Time Effort. (Bartenieff and Lewis, 1980; Bartenieff and Hackney, 1984; Bender, 2010; Kennedy, 2014).

#### Variables: The Weight Effort

The Weight Effort is about the force/pressure exerted throughout a movement or the quality of the exertion of weight, but not the body weight *per se*. The indulging quality of the Weight Effort is Light, which means a release of force/pressure throughout a movement. The fighting quality of the Weight Effort is strong, which means an increase of force/pressure throughout a movement. So, the opposite of light is not a passive heaviness but strength. For example, picking up a delicate, small object is Light, smashing an object with a fist is Strong. Jumping in ballet is Light but stamping your feet in African dance or sumo style is Strong (Bartenieff and Lewis, 1980; Foroud and Whishaw, 2006; Bender, 2010; Kennedy, 2014).

### Data Analysis

In previous studies, the reliability of the non-kinematic measures in LMA, which includes the Effort System, has already been validated (Fagen et al., 1997; Foroud et al., 2004). In this study the inter-rater reliability was tested with Cohen’s Kappa (Lausberg et al., 1996; Grouven et al., 2007; Cruz and Koch, 2012; Bühl, 2016). The inter-rater reliability was interpreted in accordance with Landis and Koch (1977).

The comparison between the groups – the burnout group and the healthy control group – was tested using the Mann–Whitney *U* test since the data was non-parametric and ordinally scaled (Levy and Duke, 2003; Bühner and Ziegler, 2009). Following the hypothesis, that there were deficits within all Effort Elements, they were tested one-tailed.

To control the influence of the following variables, they were gathered and analyzed in ordinal regression models: age and gender, educational level as well as dance affinity, dance and dance therapy experience.

The bivariate correlation between all variables of the study was tested with using Spearman’s rank correlation. The effect size was interpreted in accordance with Cohen (1988).

A power analysis was conducted *post hoc*.

Statistics were performed using SPSS 23.

## Results

### Inter-Rater Reliability

The Kappa coefficients ranged from 0.66 to 0.92 (*p* < 0.001) with the Confidence Interval (≤ 95%) ranging from 0.457 to 1.009 (see **Table 1**). Landis and Koch (1977) suggested that 0.61–0.80 can be considered as a substantial agreement, and 0.81–1.00 as an almost perfect agreement among the raters.

**Table 1:** Inter-rater reliability, confidence interval of the dependent variables (LMA).

Effort	Variables	Kappa	95% CI
Flow effort	Free	0.67	0.46–0.88
	Bound	0.89	0.77–1.01
Space effort	Indirect	0.74	0.57–0.91
	Direct	0.80	0.62–0.97
Time effort	Sustained	0.66	0.47–0.86
	Sudden/Fast	0.84	0.69–0.99
Weight effort	Light	0.75	0.57–0.94
	Strong	0.79	0.61–0.96

*Kappa, Inter-rater reliability; CI, confidence interval.*

### Burnout Patients vs. Control Group

Supplementary Table S1 shows descriptive statistics for all variables, i.e., mean and/or median; the measure of dispersion of all variables, i.e., standard deviation and/or range; Cronbach’s alpha of the Burnout Screening Scales (BOSS I–III); the correlations of all variables of the study, i.e., the variables of the LMA Effort System and the variables of the BOSS I–III. As the variables of the LMA Effort System were ordinally scaled only their median and range were calculated. As seen in Supplementary Table S1, almost all variables of the LMA inter-correlated except for a few combinations (two combinations with the variable Direct). Likewise, almost all variables of the BOSSI–III inter-correlated with a few exceptions (particularly combinations with the variables Job3, Family3). There was a negative correlation between variables of the BOSS III and variables of the BOSS I and II. Furthermore, some variables of the LMA correlate with the BOSS I and II as well as with the variable Self of the BOSS III: Bound, Indirect as well as Sustained with two exceptions, and Light with one exception (see Supplementary Table S1).

The comparison of the burnout and control groups revealed that the burnout patients have significant deficits in all four Efforts of the LMA Effort System: Flow, Space, Time and Weight Effort. In each Effort, burnout patients showed a significant deficit in comparison to the control group for one element: Regarding the Flow Effort, burnout patients showed significantly less frequent Bound movements *U*(*n*_1_ = 22, *n*_2_ = 20) = 112.5, *p* = 0.001, and less frequent Free movements, but not significantly *U*(*n*_1_ = 22, *n*_2_ = 20) = 158.5, *p* = 0.05 (see **Table 2**) in comparison to the control group. Within the Space Effort, burnout patients had significant deficits within Indirect movements *U*(*n*_1_ = 22, *n*_2_ = 20) = 114.5, *p* = 0.001, but regarding Direct movements there were no significant differences *U*(*n*_1_ = 22, *n*_2_ = 20) = 201, *p* = 0.3 (see **Table 2**). The results of the Time Effort showed that burnout patients had significant deficits within the Sustained movements *U*(*n*_1_ = 22, *n*_2_ = 20) = 130, *p* = 0.01, but there were no significant differences regarding the Sudden or Fast movements *U*(*n*_1_ = 22, *n*_2_ = 20) = 174.5, *p* = 0.11 (see **Table 2**). Finally, burnout patients showed significantly less frequent Light movements *U*(*n*_1_ = 22, *n*_2_ = 20) = 115, *p* = 0.001, but there were no deficits within the Strong movements *U*(*n*_1_ = 22, *n*_2_ = 20) = 220, *p* = 0.52 in the Weight Effort (see **Table 2**). The results showed that only within the Flow Effort did burnout patients have deficits regarding the fighting element. Within the other three Efforts – Space, Time, and Weight – burnout patients had deficits regarding the indulging element.

**Table 2:** Demographic variables, mean ranks of the Mann–Whitney *U* test (patients/control group) with significances of the dependent variables (LMA) and power.

	Burnout patients (*n* = 22)	Control group (*n* = 20)	Power^∗∗∗^ (1-β err prob)
Gender: men (%)	14 (63.6%)	10 (50%)	
Age [*M*(*SD*)]	47.2 (9.1)	41.5 (15)	
**Educational level (%)**		
Low	13 (59.1%)	4 (20%)	
Middle	5 (22.7%)	6 (30%)	
High	4 (18.2%)	10 (50%)	
Dance affinity: yes (%)	11 (50%)	12 (60%)	
Dance (therapy) experience: yes (%)	3 (22%)	8 (40%)	
Medication: yes (%)	21 (95.5%)	2 (10%)	
**Flow effort (median)**		
Free	18.7	24.58	0.2
Bound^∗∗^	16.61	26.88	0.39
**Space effort (median)**		
Indirect^∗∗^	16.7	26.78	0.38
Direct	22.36	20.55	0.08
**Time effort (median)**		
Sustained^∗∗^	17.41	26	0.3
Sudden/fast	19.43	23.78	0.15
**Weight effort (median)**		
Light^∗∗^	16.73	26.75	0.39
Strong	21.5	21.5	0.05

*^∗^p < 0.05, ^∗∗^p < 0.01, ^∗∗∗^ = post hoc.*

### Regression Analysis

Results of the ordinal regression analysis to control the variables age and gender, educational level as well as dance affinity, dance and dance therapy experience are not presented here because the requirements for the models were not fulfilled due to the limited sample size (see section “Discussion”).

## Discussion

Based on literature concerning the definition of burnout which considers exhaustion as the relevant symptom (Maslach et al., 1996; Schaufeli and Enzmann, 1998; Brenninkmeijer and Van Yperen, 2003; Bekker et al., 2005; Cox et al., 2005; Kristensen et al., 2005; Van Dam, 2016), and based on the literature about analysis of the LMA Effort System for other psychiatric diseases (Burn, 1987; Shenton, 1990; Lausberg et al., 1996; Lausberg, 1998; Welsche, 2010) as well as on the results of the pilot study, it was expected that burnout patients would show deficits in their movement regarding the LMA Effort System. It was expected that every Effort would be affected. The results confirmed that indeed every Effort was affected within burnout: burnout patients showed significant deficits in relation to Flow, Space, Time, and Weight in comparison to the healthy control group.

Regarding the Flow Effort (Free – Bound), outcomes from this study showed that burnout patients demonstrated a significant deficit concerning Bound movements, but not in terms Free movements. Following Bender (2010) or Kennedy (2014) Bound movements refer to controlled movements that can be stopped at any time. Therefore, Bound Flow leads to a control of the movement that helps to resist external stimuli, protecting against these. As a result, with Bound Flow the influence of external stimuli can be regulated. The results of our study showed that Bound Flow is significantly less frequent amongst burnout patients when compared to healthy subjects. This reminds us of the non-regulation in their flow of work: they work until they burn out, without stopping or decelerating early enough (Burisch, 2014).

Regarding the Space Effort (Indirect – Direct), burnout patients had a significant deficit of Indirect movements, i.e., they show significantly fewer Indirect movements than healthy subjects, but they did not show a deficit in Direct movements, following the outcomes from this study. Like Bender (2010) and Kennedy (2014) stated, indirect relatedness to the space does not concentrate on details but is able to perceive more things in parallel with wider attention and it is able to pursue various objectives. With this flexible orientation, obstacles can be avoided easily (Bender, 2010; Kennedy, 2014). However, this study showed that burnout patients have a deficit concerning Indirect movements and they show Direct movements by focusing solely on one object. This reminds us again of the work attitude of burnout patients: they become absorbed in one objective and get lost because of their lack of orientation toward the whole situation. If the focus of interest is removed, nothing remains – as the phase models of burnout (Edelwich and Brodsky, 1980; Freudenberger and North, 1985) shows.

Regarding the Time Effort (Sustained – Fast/Sudden), the results from this study showed that burnout patients had a significant deficit in terms of Sustained movements, i.e., they show significantly fewer Sustained movements than the healthy subjects, but they did not show a deficit concerning Sudden/Fast movements. Following Bender (2010) Sustained is not to fight against time, not to be trapped in time, but rather it is about the sensation of reveling in the run of time. However, the outcomes from this study showed that burnout patients with their deficit of Sustained movements are not able to revel in time. This reminds us of the problem of burnout patients running out of time, so they cannot revel in time in general (Burisch, 2014).

The indulging quality of the Weight Effort, Light movements refer to a release of force/pressure throughout a movement (Bender, 2010; Kennedy, 2014). Here burnout patients had a high significant deficit, like this study showed. They showed significantly fewer airy and delicate movements. This reminds us of the problem that burnout patients cannot generally take things lightly (Burisch, 2014).

Consequently, following the outcomes from this study, it seems, that burnout impairs Bound, Indirect, Sustained and Light movements although it cannot be known if this is caused by burnout. To answer this question, the body movements of burnout patients following recovery should be analyzed.

### Comparison to Literature

To the knowledge of the authors the movement of burnout patients has not been investigated yet – neither with the LMA nor with any other movement analysis system. Thus, the present study is the first in this area. Therefore, the results can only be compared with results from other psychiatric disorders not with burnout studies. In particular, the comparison with depression, anxiety and chronic fatigue syndrome seems to be interesting since the differentiation between burnout and these diseases is not clear (Korczak et al., 2010; Bianchi et al., 2015a; Van Dam, 2016). To date, there has been no investigation, to the knowledge of the authors, which has analyzed the movements of patients with anxiety or chronic fatigue syndrome, but there is one which analyzed the movements of patients with depression: Welsche (2010) found that patients with depression showed overall a smaller repertoire relative to the Effort System. Moreover, they showed a preference for the fighting Effort elements, which are Bound, Direct, Fast/Sudden and Strong.

The outcomes from this study showed that burnout patients with their deficits within all Efforts of the Effort System have a smaller repertoire relative to the Effort System, similar to patients with depression in the study of Welsche (2010). Nevertheless, in contrast to patients with depression, burnout patients do not have a preference for Bound movements but display significantly fewer frequent Bound movements – i.e., the fighting side of the Flow Effort is significantly affected amongst burnout patients. So, there is a clear difference between the findings of Welsche (2010) concerning patients with depression and the findings of this study with burnout patients. Therefore, the present findings could lead to further grounds for investigating the differentiation of burnout and depression. But there are limitations concerning this comparison (see limitations).

Bartenieff and Lewis (1980) and Bender (2010) suggest that patients with depression tend to show no Weight Effort at all, since they have a quality of a passive heaviness, and therefore neither the active Strong nor the active Light Weight Effort element. Instead, Welsche (2010) found that patients with depression showed a preference for the Strong Weight Effort element. However, burnout patients have indeed a deficit within the Light Weight Effort element, but they do not show fewer frequent Strong movements than the control group, and therefore, they demonstrated the Weight Effort quality.

### Quality Criteria of the Methods

In previous studies, the reliability of the non-kinematic measures in LMA, which includes the Effort System, has already been validated (Fagen et al., 1997; Foroud et al., 2004). In this study the inter-rater reliability, i.e., the consistency of the movement analyses (Landis and Koch, 1977; Lausberg et al., 1996; Grouven et al., 2007; Cruz and Koch, 2012; Bühl, 2016) was substantial to almost perfect. Therefore, the objectivity and reliability of the analyses are proven.

In this study the Burnout-Screening-Scales (BOSS I–III; Geuenich and Hagemann, 2014), a German burnout test, were used as a burnout questionnaire. The measurement properties of this test are good. The questionnaire shows a middle to high test–retest-reliability, so the test measures the relevant complaints reliably and without large fluctuations within a short period of time (2 days; Geuenich and Hagemann, 2014). The questionnaire shows also good criterion-related validity as the statistical values coincide with the clinical diagnosis of psychiatrists (Geuenich and Hagemann, 2014). The BOS-Scales III are about resources (Geuenich and Hagemann, 2014) – and as Jimenez and Dunkl (2017) suggested, resources should also be taken into consideration regarding burnout. Furthermore, the BOSS is not only job-related, for which the MBI has been criticized (Lehofer et al., 2011). The inter-correlations in this study demonstrated that the variables of the BOSS I–III correlated to each other. This means that the variables are homogenous. Exceptions are, in particular, the combinations with the two variables, Job3 and Family3, which are about satisfaction and resources in the areas of one’s own job and one’s own family and/or partnership (see section “Materials and Methods”). Due to the fact that BOSS III measures the resources of the participants, the variables of BOSS III correlate with the variables of BOSS I and II negatively. Additionally, the variables of the LMA correlate with each other, so these variables are also homogenous. Exceptions are two combinations with the variable Direct. Finally, there are correlations with variables of the LMA – Bound, Indirect, Sustained and Light (the latter two with some exceptions, see section “Results”) – with the variables of BOSS I and II as well as with the variable Self of BOSS III. These intercorrelations are the very first indications that movement analysis could possibly provide an extension of diagnostic advice in the future (see Supplementary Table S1).

### Limitations

Some limitations of this study need to be mentioned.

Firstly, for the study only a small number of subjects participated. As the sample size is too small the *post hoc* power analysis showed no satisfying results to statistically optimally ensure the effect.

To control the influence of the following variables in this study, they were gathered and analyzed in ordinal regression models: age and gender, educational level as well as dance affinity, dance and dance therapy experience. Age and gender were analyzed together in one regression model, as well as dance affinity and dance (therapy) experience. Due to the limited sample size the requirements for the models were therefore not fulfilled and the results of the ordinal regression analysis were not presented. However, they may be presented here: the effect was still significant despite controlling age and gender of the participants. Gender significantly influenced only the outcome of the variable Direct Space, which was not significantly affected by burnout. A comparable age-distribution among the two groups was attempted in order to avoid the influences of age related changes in movements. So elderly people (over 65 years) or non-mature/young adults (under 25 years) were excluded. The remaining age-related differences of the participants showed, as previously stated, no influences regarding the significant effects whatsoever in a regression model. One age related change in movement is of course to that of becoming slower. Here we can additionally see, that the older burnout group has deficits in terms of being slow but is not significantly different in regarding being sudden/fast regarding in relation to the control group. The effect was also still significant despite controlling the educational level. Only the outcome of the variable Strong Weight, which was not significantly predicted by burnout, was significantly predicted by the educational level. And finally, the effect was still significant despite controlling dance affinity and experience with dance or dance therapy. Dance affinity significantly predicted only the outcome of the variable Free Flow, which was not predicted by burnout. To avoid the possibility that only subjects with dance affinity participated in the study in the first place, the participants did not know in the beginning that it was a dance study.

Secondly, the intake of psychotropic drugs could influence the movement, as we know there are psychotropic drugs, such as sedatives and stimulants. Data regarding the intake of medication was collected. All burnout patients took psychotropic drugs, and none were taken by the control group. Burnout patients took sedating as well as stimulating psychotropic drugs, often together. Therefore, it cannot be controlled.

Thirdly, burnout is not recognized in the international disease classification systems (DSM-5, ICD-10) as a distinct disease, and therefore, there may be problems with the diagnosis of burnout. In this study the diagnosis was double checked by clinicians and by using the German version of the questionnaire Burnout Screening Scales (BOSS; Geuenich and Hagemann, 2014). However, one of the problems with burnout research is the continuing lack of international consensual diagnostic criteria for burnout to identify burnout subjects in the first place in order to be able to investigate burnout. So, this study could only begin at a certain point, with the knowledge of the underlying discussions and therefore also with these underlying limitations. Thus the diagnose of burnout by this investigation is limited to the diagnosis of the psychiatrists at the clinic as well as the German burnout questionnaire with good measurement properties (BOSS; Geuenich and Hagemann, 2014).

Fourthly, differential diagnostics (depression, anxiety or fatigue disorders etc.) were not tested by additional psychological tests but were implicit with respect to the psychiatric diagnoses by the psychiatrists at the clinic and the burnout questionnaire results. So, a possible comorbidity between burnout and depression – as it is discussed in literature (Ahola et al., 2005; Bianchi et al., 2014, 2015b) – was not clarified by means of additional psychological tests. Therefore, a comparison of the outcomes from this study with studies concerning depression (Welsche, 2010) can only be treated with caution.

Fifthly, in line with the phase models of burnout (Edelwich and Brodsky, 1980), there were subgroups regarding the degrees of severity amongst the burnout patients, who cannot be diagnosed by the normal diagnosis system, (Van Dam, 2016) and their different speeds of recovery, so we cannot guarantee homogeneity amongst the burnout patients. Although hospitalization, current inability to work, the diagnosis of a clinician and the Burnout Screening Scale (BOSS; Geuenich and Hagemann, 2014) and time after hospitalization were comparable.

## Conclusion

The present findings can give additional indications for burnout: Burnout patients have deficits regarding the Efforts in their movement. They had a significantly smaller repertoire relative to the LMA Effort System, Bound, Indirect, Sustained, and Light movements are particularly affected. Consequently, burnout patients showed deficits in their body movements.

The findings can also give – with the LMA Effort System – new starting points for therapeutic interventions for burnout, i.e., specific movement programs.

Further research is advised: we should investigate if the movements, and especially the Efforts, are also affected after the burnout patients have recovered and to answer the question whether the affected Efforts are a consequence of burnout or if subjects with affected Efforts usually tend to suffer from burnout. Moreover, different psychiatric disorders should be analyzed and compared with burnout in one study. Finally, the impact of therapeutic interventions for burnout on the basis of the LMA Effort System should be investigated.

## Ethics Statement

All procedures performed in studies involving human participants were in accordance with the ethical standards of the institutional and/or national research committee (GZ. 39/5/63 ex 2014/15, 08.01.2015) and with the 1964 Helsinki Declaration and its later amendments or comparable ethical standards.

## Author Contributions

MP designed the study, analyzed and interpreted the data, and drafted the article. AP did the original conception of the study and revised in all steps. GS acquired the participants.

## Conflict of Interest Statement

The authors declare that the research was conducted in the absence of any commercial or financial relationships that could be construed as a potential conflict of interest.
